# Editorial

**Published:** 1915-11

**Authors:** 


### EDITORIAL

The Atlanta Journal-Record of Medicine has its Business Office at 23 West Alabama Street where all business communications should be sent.

Remittances are payable to The Journal-Record of Medicine.

Concerning Original Communications and Editorial matters, address Richard R. Daly, M. D., 708 Empire Life Building, Atlanta, Ga.

Reprints of Original Articles will be furnished at cost price. Requests for them had best be made on the Manuscript.

Upon request, we will present, postpaid, to each contributor of an original article, twenty copies of The Journal-Record of Medicine containing such article.

### THE "CHICAGO" BABY.

The newspapers have all been mentioning the situation a Chicago doctor found himself in when a mother whom he confined, presented him with a monstrosity that was alive and had a sort of chance to continue to live if some operation was performed. After consultation with the family and others supposedly qualified to give advice, the doctor left the operation undone and the infant died.

Later examination by the coroner disclosed to the public that the child was minus an ear and part of a neck and that its general make-up was a useles jumble of many things found in a child.

The editorial cogitations of the newspapers have been interesting quite as much from the positiveness with which they have taken opposite stands as for anything else. Some consider the opening it might leave for the doctor to do unlimited murder if this negative act had general approval, while others endeavor to show that no one can know the outcome of the growth of a being seemingly impossibly handicapped and they point out the recoveries of men who have been given up to die by the doctor, in support of this.

These two parts appeal to us. Of course everyone deep down in his heart as they used to say at Sunday School, and may yet perhaps, everyone knows that the fundamental tendency and desire, the pinnacle of the ambition of every doctor is to do murder and that he has been waiting all these years for the smallest of openings through which he can drive the entering wedge of the privilege of killing whom he may without punishment therefor. It is fitting that he begin with a slaughter of the innocents because they are easier to get at and less resistant. . The ordinary human being, outside whose pale the doctor is by nature created and reared, has a penchant for loving and petting babies and making the most of their charming society. Men have been known to boast to their business associates of the beauty of their daughters and the lustiness of their sons when they—the children—were extremely young. But the doctor is different. He is gleeful when a child is to be born. He sees not only an accouchement fee, but another chance to kill somebody and to set the practice up in safety for his fellow ghouls.

There are some doctors who have so far covered their real nature with a cloak of humanity that they have accepted invites from intelligent mothers to act as experts and judges in Better Baby Shows so that the finest of children as well as those who are ailing, may have attention given to little matters not likely to be discovered at home, and advice given to the mothers as to the future care. But these horrid men are studying the beautiful, little, naked bodies brought before them with epicurean eyes and palates, too, let us say, because some day they can do as they like with them.

The man who loses sleep and risks carrying diptheria infection to his own children—and here doctors again imitate humans in having children—the man who takes risks working at the side of the child that will die without his care is doing it regretfully or else because he does not believe in "natural" death. The men who put the antitoxins among the therapeutic agents must have had some ulterior motive which the news editors can discover better than we know.

So don't trust the doctor to do what is right under such circumstances as appalled the Chicago practitioner. He has no human instinct and his training is all wrong.

As to the future course of life, we are as little prophetic as our neighbors and the man who claims to be more so comes to grief as soon as the cards turn or some one gets up and walks twice around a chair. But, and here we do come in,—but we can recognize what we are looking at and say with confidence that if a child has no ear on one side, there is no ear on that side; and if his cheek is grown fast to his shoulder on the other side, it is fast there; and if the skull is too small and flat for a full-sized brain, the full-sized brain must be somewhere else than in the skull. Then, having these facts before us after the arduous labors necessary to collate them, we still have energy enough to suspect that since we have never seen or heard of the spontaneous development of these organs postpartum, we are right in continuing to suppose that no miracle is to be performed in our behalf and that these defects will not be remedied. Even so, shall the child live? Well, we have noticed that barring some things we never could comprehend and upon which newspaper editors on medical and surgical topics are doubtless better informed, a growing child and an adult individual need these things to get along as a human being should. That is apparent perhaps until it comes to the scarcity of cephalic space. We cheerfully agree that megalocaphaly is not always associated with brilliancy and achievement especially when there has been water with it. Cogent reasoning might take us to the other end of the string and show that pinheadism was the ultimate goal of the genious-producing families, but the doctor looking at a monstrosity has to deal only with facts and he can't figure this all out every time.

He let the child die and he did no murder. He was wise.

The question was asked of a large medical society what its members would advise doing under the circumstances. There were but two or three answers and these were not decisive. The men knew the problem was a big one and that each had for itself peculiarities that would forbid a general rule applying to it. Each man knew also that there were many elements entering into the solution of every such case that has come before him which none but the family doctor can know and that these had to be considered and weighed together with the child's condition. Each man knew what his responsibility would be and having been trained in matters of life and death from the days of his youth, he knew that he could trust himself to do what he thought right when cases presented themselves and that there was no need of a rule and exceptions.

We know what the various religious teachings are as to this affair, but, well, when the religions of the world have complete control of the conceptive end of foetal life, it will be time enough to yield everything to them at the beginning of extra maternal existence or at the beginning of everything else, for that matter.

---

### IN FAIRNESS.

In our September issue, Gray's Glycerine Tonic Comp. was inadvertently included in a list that seemed to be under the ban of the Government and very likely an injustice has been done the Purdue Frederick Company which we desire to undo as far as possible.

In our advertising columns we put forth only those firms and those products that are so far as we know, reputable and worthy of the consideration of our readers. We do not attempt to go beyond this and advise as to whether the articles shall be used or not, for we are speaking to a clientele peculiarly able to judge for itself after their attention has been called to the matter.

We are entirely independent of outside influence either from those who offer us fees for advertising or from those who are not qualified to give us advice as to the conduct of our advertising space. We do desire to please our subscribers and to place before them what it is likely they may wish to buy and use. In doing this we attempt to keep in close touch with the common sense of the medical profession and always to play fair.

To the basic principles of medical ethics, we are hearty subscribers. They may be summed up with the terms common decency and fair play, or if there are any outside of this, we are not familiar with them.

W note that in the Federal prison in Atlanta, the warden has instituted a "Father-Adviser Day," during which he sees any prisoner who has applied for the interview and counsels with him upon any subject the prisoner may introduce. Thus at least once a week, the man highest up may be seen confidentially and injuries remedied, misunderstandings cleared and hopes revived and stimulated.

This is in line with the awakening sentiment of the people who have gained knowledge of the criminal and have seen the light that after all he is pretty much like anyone else and that he does not cease to be human because he has done something anti-social and been caught at it. It is also in line with the conclusions of observant people that punishment does no good unless it protects one from doing further wrong and warns others to refrain from injurious conduct. Punishment per se is destructive, no matter one looks at it, and the apparent victim is not the worst sufferer. The man and the group of men who take vengeance in that way have nailed the limits of their minds within small compass and shut of their privilege of broad contact with the human sympathy of the world. And when that sympathy and comprehension of the other man's need fails, then civilization has no hope—no right to exist.

In the little book reviewed in this issue a wonderful picture has been drawn and a light shed upon the prison question that every thinking man especially a doctor who has power for the prevention of crime and for leading of public sentiment along humane lines should take as a guide to further study of this great question.

---

The Cincinnati Sanitorium of College Hill, Cincinnati, announces the completion of a new building which is to be devoted to the care of cases suffering with nervous and nutritional diseases proper. This shows a specialization in the care of nervous and mental diseases that will be advantageous to the patients and convenient to the physicians in charge. The separation of patients into small groups requiring each their own attention makes for success in all hospital work and removes confusion from the scene. New and well equipped small buildings are often much better than special wards in a larger place and in this instance the cottage idea has been well worked out.

---

### PREPAREDNESS.

The two articles in this number of the JOURNAL that come from surgeons in Europe show conditions on both the east and the west sides of the battlefields. One is from close to the line and the other is distinctly a base hospital report, but thy have this in common: There is so much work and so many injuries that the present generation have never seen before that a sense of profound confusion comes to the mind and we can begin to see what an intense fatigue workers must feel when a never ending line of injured is coming to them.

The men were unable to do many things because they were tired and because they did not have proper materials to do with. They were overwhelmed with wounded and had no proper space for them. As one of them puts it, he was laterally wading in blood at times.

It all sounds horrible and awful, but it is only a small part of the war and shudders are useless.

What are we going to do about it? War is on and it is imminent. That it may spread to our own country has been the fear of all men this last year. If it comes, are we as physicians, ready?

We have not the least idea of what we should do for field and base hospitals nor how we could fill up and equip a staff of competent men. We have the materials, saving the drugs that the war has deprived us of, but we have not the organization. It is true that the military authorities have some plans regarding the matter, but are they any more extensive than the piti fully small preparations for defense that some pacifists would rely upon? Once more the medical societies are asked to consider this matter.

